# Misconceptions about fructose-containing sugars and their role in the obesity epidemic

**DOI:** 10.1017/S0954422414000067

**Published:** 2014-06

**Authors:** Vincent J. van Buul, Luc Tappy, Fred J. P. H. Brouns

**Affiliations:** 1 Maastricht University, Faculty of Health, Medicine and Life Sciences, Department of Human Biology, PO Box 616, 6200MD, Maastricht, The Netherlands; 2 University of Lausanne, Department of Physiology, 7 Rue du Bugnon, CH-1005, Lausanne, Switzerland

**Keywords:** Fructose, Sugar, Sugar-sweetened beverages, Obesity

## Abstract

A causal role of fructose intake in the aetiology of the global obesity epidemic has been proposed in recent years. This proposition, however, rests on controversial interpretations of two distinct lines of research. On one hand, in mechanistic intervention studies, detrimental metabolic effects have been observed after excessive isolated fructose intakes in animals and human subjects. On the other hand, food disappearance data indicate that fructose consumption from added sugars has increased over the past decades and paralleled the increase in obesity. Both lines of research are presently insufficient to demonstrate a causal role of fructose in metabolic diseases, however. Most mechanistic intervention studies were performed on subjects fed large amounts of pure fructose, while fructose is ordinarily ingested together with glucose. The use of food disappearance data does not accurately reflect food consumption, and hence cannot be used as evidence of a causal link between fructose intake and obesity. Based on a thorough review of the literature, we demonstrate that fructose, as commonly consumed in mixed carbohydrate sources, does not exert specific metabolic effects that can account for an increase in body weight. Consequently, public health recommendations and policies aiming at reducing fructose consumption only, without additional diet and lifestyle targets, would be disputable and impractical. Although the available evidence indicates that the consumption of sugar-sweetened beverages is associated with body-weight gain, and it may be that fructose is among the main constituents of these beverages, energy overconsumption is much more important to consider in terms of the obesity epidemic.

## Introduction

Several studies and reports^(^ ^1^ ^–^ ^8^ ^)^ have indicated an increased consumption of sugar-sweetened beverages (SSB) over the period 1970–2005 in the USA and Europe. The SSB category includes sodas (soft drinks), fruit drinks, sports drinks, ready-to-drink sweetened tea and coffee, rice drinks, bean beverages, sugared milk drinks, sugar cane beverages and non-alcoholic wines or malt beverages. The increased intake was related to a high availability of such products in the market, amplified marketing efforts, and larger portion sizes, which increased three- to five-fold over time^(^ ^9^ ^)^. As such, SSB consumption was suggested to be a considerable amount of total daily energy intake^(^ ^10^ ^)^.

Interestingly, over the last 5 years, the global annual consumption of carbonated soft drinks has remained constant or even has declined^(^ ^11^ ^)^, while bottled water has increased to more than 1 litre per individual per year in recent years^(^ ^12^ ^)^. Obesity rates, however, seem to have increased independently of these shifts in beverage intake^(^ ^13^ ^)^. An overview of the average Western European consumption of the five most common drink categories, including SSB, per capita per year are given in Table 1 ^(^ ^14^ ^)^. Data from the USA are given in Table 2 ^(^ ^15^ ^)^. Note that in the USA, carbonated water is replaced by sports drinks in the five most consumed categories.

**Table 1:** Average Western European consumption of the five most common drink categories, including sugar-sweetened beverages^(^ ^14^ ^)^

	European average yearly consumption (litres per person)
Drink category	2007	2008	2009	2010	2011	2012
Still bottled water	54·0	55·2	55·6	56·3	57·5	58·5
Cola carbonates	28·4	28·7	29·4	30·1	30·7	31·0
Carbonated bottled water	28·2	27·4	27·2	27·1	27·4	27·7
Non-cola carbonates	20·7	20·6	20·8	20·9	20·9	20·9
100 % Juice	12·9	12·5	12·1	11·9	11·5	11·2

**Table 2:** Average US consumption of the five most common drink categories, including sugar-sweetened beverages^(^ ^15^ ^)^

	US yearly average consumption (litres per person)
Drink category	2007	2008	2009	2010	2011	2012
Still bottled water	70·3	68·0	66·5	66·2	68·2	70·9
Cola carbonates	71·5	67·9	65·0	62·8	59·5	56·5
Non-cola carbonates	56·1	54·9	54·0	54·9	55·7	55·9
Sports drinks	16·3	15·4	13·9	14·8	17·0	17·3
100 % Juice	16·5	16·4	16·4	15·6	14·8	14·3

Although data from Table 1 and Table 2 were obtained through trade sources and national statistics (by Euromonitor International^(^ ^14^ ^,^ ^15^ ^)^), which did not account for wastage and were not corrected for export to other countries, it can be concluded that, even if intake patterns are shifting, consumers in different parts of the world still purchase a relatively high amount of SSB.

In this light, the systematic reviews by Malik *et al.* ^(^ ^16^ ^)^ in 2006 and Hu & Malik^(^ ^17^ ^)^ in 2010 concluded that such quantities of SSB consumption were associated with both weight gain and type 2 diabetes prevalence. Moreover, results from a survey in Australia indicated that high SSB intake may be an important predictor of cardiometabolic risk^(^ ^18^ ^)^. A scientific opinion by the European Food Safety Authority (EFSA)^(^ ^19^ ^)^, however, concluded that additional justification for the correlation between SSB consumption and such adverse health effects was required.

In this respect, one may question what in SSB could be responsible for these adverse effects on health^(^ ^20^ ^)^. More specifically, first, is there evidence that specific sugars, such as fructose and glucose, as present in sucrose and high-fructose corn syrup (HFCS), promote excess energy intake? Second, is there evidence that excess energy intake as sugars is more detrimental to health than excess energy as fat, or as complex carbohydrate present in potatoes, rice, refined cereals, and so forth?^(^ ^21^ ^)^.

Since the recent publications of Lustig and co-workers^(^ ^22^ ^,^ ^23^ ^)^, in which it was suggested that fructose is toxic and should be ‘treated as alcohol’, the daily news all over the world highlighted fructose in SSB as a potential poison. It was proposed that fructose is a causal factor in obesity aetiology, based on the scientific evidence that substantiated that fructose, when consumed in excessive amounts, led to detrimental effects on body-weight regulation, lipid metabolism and glucose homeostasis in animals and in human subjects^(^ ^24^ ^–^ ^27^ ^)^. As a result, an overall reduction in the global consumption of fructose-containing sugars was recommended in recent literature^(^ ^1^ ^,^ ^4^ ^,^ ^16^ ^,^ ^28^ ^–^ ^36^ ^)^. To achieve this reduction, various measures have been proposed^(^ ^37^ ^)^, most of which related to extra taxes on foods, such as SSB, that are considered unhealthy because of their high fructose content^(^ ^38^ ^–^ ^43^ ^)^. However, raising tax levels, and consequently purchase prices, has generally failed to change consumption behaviours^(^ ^44^ ^)^. In line with this, also the removal of products from the site of availability has been discussed as possibly inappropriate in changing purchase behaviour, since it may result in exchanging the purchase with similar products^(^ ^45^ ^)^. In the field studies of Wansink *et al.* ^(^ ^45^ ^)^, it was evidenced that taxing soft drinks in Utica (New York State) led beer-buying households to increase their purchases of beer. Similarly, taking out snacks and soft drinks from vending machines did not withhold children from buying such products at other locations or finding other alternatives that are also high in sugars, fat, and energy-dense.

Moreover, as the human body does not differentiate fructose absorption, whether it comes from HFCS, cane or beet sugar, or from an intrinsic source such as that present in fruits or fruit juices^(^ ^19^ ^,^ ^46^ ^,^ ^47^ ^)^, would this reduction also be necessary for fruits that contain relatively large amounts of fructose such as apples, apricots and ripe bananas? Should honey also be removed from our diet^(^ ^48^ ^)^? These questions have confused the typical consumer of sweet (and sweetened) food products^(^ ^49^ ^)^. This confusion may have been intensified by the issuing of a scientific opinion on fructose by EFSA in 2011. With this, European food manufacturers can claim that ‘consumption of fructose leads to a lower blood glucose rise than consumption of sucrose or glucose’^(^ ^50^ ^)^. Having evaluated the scientific literature at their disposal^(^ ^47^ ^,^ ^51^ ^–^ ^57^ ^)^, the EFSA panel assumed that, when fructose replaces sucrose or glucose in foods or beverages, the claimed effect will be obtained. The panel took into account two human intervention studies^(^ ^51^ ^,^ ^52^ ^)^ that showed a consistent significant reduction in postprandial glycaemic responses. This occurred without disproportionally increasing postprandial insulinaemic responses. Further, the panel noted that the mechanism by which fructose (when replacing sucrose or glucose) in food or beverages could exert the claimed effect was well established.

The panel did note that high intakes of fructose (set at ≥ 25 % of total energy) was shown to lead to metabolic complications such as dyslipidaemia, insulin resistance and increased visceral adiposity, based on several review articles^(^ ^47^ ^,^ ^53^ ^–^ ^56^ ^)^. With this scientific opinion, and related health claim, the panel clearly took a different position from the opinion that fructose is toxic and should be treated as alcohol.

So, what is the current status concerning the role of fructose-containing SSB that supply glucose along with fructose? Identifying added fructose as a prime cause of obesity can be misleading to the public, as well as policy makers, about the ‘truth of obesity’ in the case that causality remains unproven. Obesity is recognised to be a multiple-factor-related health problem^(^ ^58^ ^)^, in which lifestyle factors^(^ ^59^ ^)^, eating behaviour^(^ ^60^ ^)^ and socio-economic aspects^(^ ^61^ ^)^ all play a key role, and fructose intake may be just one among several factors involved in its prevalence. At present, there are reasons to believe that isolated reductions in added fructose-containing sugar intake, as recently investigated^(^ ^62^ ^,^ ^63^ ^)^, will not lead to a decrease in obesity prevalence. When similar isolated reductions were undertaken concerning added fats^(^ ^64^ ^)^, the desired overall reduction in fat intake and development of low-fat/light products were not observed^(^ ^65^ ^)^.

Fructose is considered by some authors to be a significant culprit for obesity and related disorders based on three categories of arguments:

(1) Arguments that generalise data derived from animal models of obesity (in which sugar overfeeding was used as an experimental tool to increase body weight) as well as human studies in which excessive fructose intakes were used to study the mechanisms of metabolic dysregulation.
(2) Arguments that confuse the relative contents of glucose and fructose in industrially produced food and beverages.
(3) Arguments that underestimate our personal responsibility to remain physically active and to consume a healthy diet.

A plethora of unbalanced reviews on the topic have recently been published^(^ ^23^ ^,^ ^66^ ^,^ ^67^ ^)^, including citations to other reviews instead of addressing the authentic data. In the present review, we therefore look at evidence regarding both positive and negative effects of fructose and fructose-containing sugar sources on obesity, as described in recent peer-reviewed research papers.

## Metabolic effects of fructose

In order to study the effects of fructose on metabolism, scientists have generally used dosages high enough to observe some significant effects, mostly in animal studies and sometimes in human intervention research. Based on recent publications^(^ ^27^ ^,^ ^56^ ^,^ ^68^ ^,^ ^69^ ^)^, we summarise a number of key findings from studies with high to excessive fructose intakes. It is important to note that fructose intake varies between individuals, based on their daily consumption patterns^(^ ^70^ ^)^. Through a 2008 US survey in 21 483 children and adults, it was found that the mean intake of fructose was 9·7 (standard error of difference 0·1) % of total energy intake, and that 95 % of these sampled individuals consumed less than 19·5 (standard error of difference 0·7) % of fructose as part of their total energy intake^(^ ^70^ ^)^. Therefore, in the discussion below, we assume fructose intake to be excessive if its pure intake amount is larger than 20 % of daily energy.

### Effects of excessive doses of fructose

Already in 1993, researchers^(^ ^71^ ^)^ agreed that excessive fructose consumption (then defined as 7·5 % to 70 % of total energy intake) induces immediate *de novo* lipogenesis in both animals and humans, because, in different experimental settings, it circumvented substrate inhibition feedback mechanisms that are present for glucose when it enters glycolysis. It was shown that the dietary fructose fraction not converted to lactate in the intestinal epithelium was rapidly taken up by the liver, where it was subsequently converted first into fructose-1-phosphate, and then to triose-phosphate and pyruvate/lactate. These are both potential substrates for liver glycogen synthesis and for fatty acid production, leading to an increased TAG release from the liver into blood. Also, it was found that fructose stimulated key lipogenic enzymes by activating sterol regulatory element-binding protein-1c (SREBP-1c) in the livers of mice^(^ ^72^ ^)^.

In addition, it was found that high fructose loads (50 % of total diet) led to an increase in PPARγ co-activator 1α and 1β (PGC-1α and PGC-1β) which promoted insulin resistance and lipogenesis^(^ ^73^ ^,^ ^74^ ^)^, as well as decreased insulin receptor activation and insulin receptor substrate phosphorylation^(^ ^75^ ^)^. Subsequently, lipogenesis induced by this high fructose load was associated with the formation of larger fat deposits in adipose tissue and muscle, in animal models^(^ ^73^ ^,^ ^74^ ^)^. However, to the best of our knowledge, there are no results of long-term human intervention studies available in which comparable quantities of fructose were investigated. One short-term intervention study (96 h) examined the effects of 50 % excess energy as fructose, sucrose or glucose, and indicated that, even under these drastic conditions, *de novo* lipogenesis remained a minor pathway for fructose disposal in both lean and obese women^(^ ^76^ ^)^.

Hyperuricaemia may occur as a consequence of rapid fructose entry from portal blood into the liver, where fructose will reduce the total adenosine nucleotide (TAN) pool in liver cells. A degradation of hepatic TAN will result in the production of uric acid. In a within-subjects intervention, this was measured in obese men and women where pure fructose intake provided 30 % of total energy intake^(^ ^77^ ^)^. Chronic hyperuricaemia was also proposed to act as a promoter of insulin resistance and type 2 diabetes development^(^ ^78^ ^)^. Based on recent findings from *in vivo* research in fructose-fed rats, it was suggested that uric acid may impair the action of insulin by decreasing insulin-mediated muscle vasodilatation^(^ ^79^ ^)^. In addition, it may possibly act as an intracellular mediator to enhance hepatic *de novo* lipogenesis^(^ ^80^ ^)^. It remains unclear if these metabolic consequences can occur in humans considering moderate fructose intake level and complex dietary composition. We will discuss this in detail below.

In older adults consuming fructose daily through SSB, fructose led to stressful conditions in hepatocytes^(^ ^81^ ^)^ resulting in the release of TNF-α, a strong pro-inflammatory messenger involved in insulin resistance development^(^ ^82^ ^)^. Also in rats, excessive fructose intake (>62 % of total energy) induced oxidative stress, mitochondrial and endothelial dysfunction, resulting in hypertension^(^ ^83^ ^)^.

In summary, it appears that excessive fructose intake can have deleterious metabolic effects in both animals and humans.

### Disputable interpretations

In contrast to these deleterious effects observed in animal models and in human trials with excessive intakes, the metabolic effects of fructose presented in ordinary human diets remain poorly investigated and highly controversial. The assumption that fructose was directly involved in the occurrence of obesity relied on correlation data between the increase in HFCS consumption and obesity prevalence in the USA. This assumption has been considered as misleading for several reasons.

First, the correlation of HFCS and obesity data only happened in North America. In Europe, there was also an increase in obesity prevalence during the same period, but HFCS was not consumed to any significant amount. Moreover, the term HFCS often led individuals to believe that it had a very high fructose content. In fact, the relative proportion of fructose to glucose in HFCS 55 (55 % fructose; used in most soft drinks) and HFCS 42 (42 % fructose; mostly used in non-beverage applications) is not that different from sucrose (50:50 %)^(^ ^46^ ^)^, although absolute levels as analysed in drinks may vary. In this respect, free fructose content in sucrose-sweetened acid-containing beverages, such as colas, was found to be increased during storage due to acid-induced sucrose hydrolysis^(^ ^84^ ^,^ ^85^ ^)^.

A prospective cohort study^(^ ^86^ ^)^ indicated that higher consumption of SSB was associated with a higher risk of CHD. Additionally, a cross-sectional study^(^ ^34^ ^)^ and two other cohort studies^(^ ^87^ ^,^ ^88^ ^)^ positively associated a reduction in SSB consumption with a reduction of disease risk factors such as elevated blood pressure or weight gain. It should be mentioned, however, that relevant intervention studies with such risk factors as end-point are lacking. Interestingly, four large cohort studies showed no relationship between moderate sugar intake and type 2 diabetes^(^ ^89^ ^–^ ^92^ ^)^. The question of whether the aforementioned effects are really caused by fructose can therefore not be answered by the observational data since these show associations, not causality.

In this respect, it is important to note that through analysis of the same set of data, a positive association between obesity risk and SSB intakes was found without adjustment for total energy intake^(^ ^93^ ^)^. These outcomes from the modelling analyses may indicate that SSB consumption was not associated with obesity risk if potential impact of total energy intake was accounted for. In this light, a meta-analysis^(^ ^93^ ^)^, a descriptive time-series study^(^ ^94^ ^)^ and a cohort study^(^ ^95^ ^)^ did report a relationship between sugar intake or SSB intake and diabetes, dyslipidaemia and cardiometabolic risk factors. In all these studies, however, the relationship disappeared when the analysis was adjusted for body weight, strongly suggesting that obesity rather than sugar intake may be the factor associated with the disease status or biomarkers mentioned.

Goran *et al.* ^(^ ^96^ ^)^ did find that diabetes prevalence was 20 % higher in European Union (EU) countries with a higher availability of HFCS, as compared with countries with low availability. The authors stated that these differences were retained after adjusting for country-level estimates of BMI, population and gross domestic product. An analysis of the study, however, shed an interesting light on the reliability of these findings. The cited HFCS consumption data for the EU countries were, in fact, not consumption data at all but rather production data. In the EU, HFCS travels freely across EU borders and can thus be consumed anywhere. For instance, the article stressed that Hungary consumed significant amounts of HFCS and also showed a higher prevalence of diabetes^(^ ^97^ ^)^. In reality, most HFCS from Hungary, which was one of Europe's leading producers of this ingredient, was exported^(^ ^98^ ^)^. Consumption and production figures are, as such, two entirely different things. Even if export and import figures were accounted for, food spoilage (which can be up to 30 %^(^ ^99^ ^)^) makes a serious impact on the above findings. This is also the case in many other epidemiological research papers that have used sugar production or disappearance data as the bases for correlations with obesity, as well as papers that cite such data for building their arguments. More recently, Basu *et al.* ^(^ ^100^ ^)^ used food supply data from the UN FAO to capture the market availability of different food items worldwide. From this, the authors concluded that an increase in sugar availability was associated with higher diabetes prevalence after testing for potential selection biases and controlling for other food types, total energy intake, overweight and obesity, period effects, and several socio-economic variables such as ageing, urbanisation and income. As discussed, the market availability of food is a debatable indicator for sugar consumption.

In this respect, a recent *New York Times* article by Strom^(^ ^101^ ^)^ pointed out that, due to incorrect methodology, as discussed by Muth^(^ ^102^ ^)^, US sugar consumption in recent years has been overestimated by >20 %. Interestingly, the author implied that sugar consumption has not risen substantially since the 1980 s. This makes many assumptions based on higher production or per capita consumption data unsubstantiated. In addition, data obtained from the US National Health and Nutrition Examination Surveys, in 2005–2010^(^ ^21^ ^)^, concluded that total energy from added sugars remained rather constant, or even declined in some segments of the population, in recent years. Moreover, the consumption of added sugar through beverages contributed to only one-third of total added sugar intake, indicating that the energy from added sugars mostly came from foods rather than beverages.

### Alternative and balancing views

In animal models, excessive-fructose diets lead to hyperphagia, obesity and the development of a metabolic syndrome^(^ ^27^ ^)^. In human subjects, however, evidence is scarce. Short-term studies that used large amounts of fructose have led to relatively modest changes in metabolic profile (including hypertriacylglycerolaemia) and a moderate decrease in hepatic insulin sensitivity and no change in whole-body/muscle insulin resistance^(^ ^47^ ^,^ ^103^ ^,^ ^104^ ^)^. This may suggest that there is a large metabolic plasticity in response to dietary changes and what we observe are minor adjustments of metabolic pathways rather than pathogenic events.

There has been no evidence that relatively high levels of SSB consumption could be associated with obesity, diabetes or cardiometabolic risk in professional athletes who usually consume SSB as energy and dehydration drinks. On the other hand, there is evidence that physical inactivity, even within a few days, causes insulin resistance and dyslipidaemia in normal healthy individuals^(^ ^105^ ^)^. In this regard, two randomised within-subjects studies in healthy males and females showed that higher plasma TAG concentrations, induced by a high-carbohydrate diet, were completely prevented by physical activity^(^ ^106^ ^,^ ^107^ ^)^. Thus, the metabolic consequences of a high mixed glucose–fructose intake can be significantly modulated by exercise. In a narrative review^(^ ^108^ ^)^, it was reported that high fructose consumption induces insulin resistance, impaired glucose tolerance, hyperinsulinaemia, hypertriacylglycerolaemia and hypertension in animal models. The data in human subjects, however, were considered less clear. In this respect, fructose consumption, even in large amounts (17 % of total energy), did not result in significant effects in healthy males but did cause these effects in healthy women^(^ ^109^ ^)^. Moreover, such fructose consumption did not stimulate *de novo* lipogenesis in premenopausal women^(^ ^110^ ^,^ ^111^ ^)^. In a review addressing sugars, insulin sensitivity and the postprandial state^(^ ^112^ ^)^, it was concluded that research on animals, particularly rodents, has shown a clear and consistent effect of high-sucrose and high-fructose diets in decreasing insulin sensitivity. Again, it was underlined that experiments in human subjects have produced very conflicting results, as there is only limited evidence from human consumption data, using fructose levels of higher than 15 % of daily energy intake, for such an effect on insulin sensitivity.

### If it is not fructose, is it just added sugars in a solution?

The suggestion that HFCS is causal to obesity^(^ ^32^ ^,^ ^113^ ^)^ cannot explain why overweight and diabetes have also increased over the past decades in regions where HFCS is not (or is hardly being) used in soft drinks (for example, Europe and India), or where SSB consumption is limited (Asia and Africa)^(^ ^114^ ^)^.

Several reviews and position papers have proposed that SSB are causally related to obesity because energy-containing liquids do not elicit the same satiety signals as energy-containing solid foods^(^ ^17^ ^,^ ^115^ ^–^ ^118^ ^)^. This hypothesis was partially supported by studies that showed that supplementation with SSB increased body weight, and thus that the intake of energy from other sources was not adequately suppressed^(^ ^62^ ^,^ ^63^ ^)^. In such studies, however, the cumulated weight gain observed was substantially lower than expected from added SSB energy, indicating that there was at least partial compensation^(^ ^117^ ^,^ ^119^ ^)^. This compensatory effect and other problems in this research area were highlighted recently by Allison^(^ ^120^ ^)^ and quantified by Kaiser *et al.* ^(^ ^20^ ^)^. In their meta-analysis^(^ ^20^ ^)^, the observed weight gain from six randomised controlled trials in which the effect of SSB on weight gain was tested was compared with the theoretical weight gain in these studies. It was found that the observed data were, on average, 85 % lower than the theoretical weight gain, indicating a high compensation effect. In other words, the effect of added sugars on weight gain was much smaller than the theoretically assumed result. This does not mean that a frequent consumption of SSB does not make an impact on weight gain. It does show, however, that other factors do contribute significantly as well.

In a recent cross-sectional study, it was reported that US adolescents, who consumed high amounts of added sugars (20–30 % of total energy), had higher blood cholesterol and TAG compared with low sugar consumers (10–20 % of total energy)^(^ ^121^ ^)^. High sugar consumers had similar body weight and total energy intake compared with low sugar consumers, but a lower intake of energy from fat and protein, indicating that sugar intake was at least partially compensated^(^ ^121^ ^)^. Several smaller studies^(^ ^122^ ^,^ ^123^ ^)^ documented that liquid sugar preloads significantly reduce spontaneous food intake at subsequent buffet meals and that fructose was as efficient as glucose – in some instances even more efficient – in this regard.

Thus, these data provide a basis for arguments against the hypothesis that fructose-containing liquids have a different effect on satiety, since all energy-containing beverages seem to have similar effects^(^ ^124^ ^)^. This has led Moran^(^ ^125^ ^)^ to conclude that results have been inconsistent and that particular findings concerning the effects of fructose on satiety appear to depend on the timing, eating context and volume of preload relative to the test meal. Another study^(^ ^126^ ^)^ listed the effect of fructose on body weight in controlled feeding trials. Herein, the authors concluded that fructose does not seem to cause weight gain when it is substituted for other carbohydrates in diets providing similar energy content. In this respect, the question arises whether consuming energy through beverages results in fewer satiety signals compared with energy from solid foods. To answer this, the US 2010 Dietary Guidelines Advisory Committee^(^ ^127^ ^)^ reviewed the literature and concluded: ‘A limited body of evidence shows conflicting results about whether liquid and solid foods differ in their effects on energy intake and body weight, except that liquids in the form of soup may lead to decreased energy intake and body weight.’

Most recently, Page *et al.* ^(^ ^128^ ^)^ performed a study on neurophysiological factors that might underlie associations between fructose consumption and weight gain. For this purpose, twenty healthy adult volunteers underwent two MRI sessions at Yale University in conjunction with fructose or glucose drink ingestion in a blinded, random-order, cross-over design. The authors concluded that glucose but not fructose ingestion reduced the activation of the hypothalamus, insula and striatum – brain regions that regulate appetite, motivation and reward processing. Glucose ingestion also increased functional connections between the hypothalamic–striatal network and increased satiety. The disparate responses to fructose were associated with lower systemic levels of the satiety-signalling hormone insulin and were not probably attributable to an inability of fructose to cross the blood–brain barrier into the hypothalamus, or to a lack of hypothalamic expression of genes necessary for fructose metabolism. The authors discussed a number of limitations of this well-designed study, but did not consider the possibility that the observed effects were merely mediated by hyperinsulinaemia present after glucose, but not after fructose, ingestion. They also did not discuss that, in real life, fructose is never consumed as a single carbohydrate source but always together with glucose. Thus, dietary intakes of sucrose and HFCS all raise insulin levels significantly, and should not induce the observed brain responses to feeding fructose alone.

## Fructose and obesity

As discussed, it is generally believed that the consumption of fructose leads to an immediate increase in lipid synthesis in the liver and a subsequent increase in circulating TAG. This assumed relationship between fructose, lipid synthesis and hypertriacylglycerolaemia has been extrapolated to obesity^(^ ^28^ ^,^ ^32^ ^)^. However, careful studies in human subjects, using stable isotopes, do not confirm this relationship. Chong^(^ ^129^ ^)^ observed that, after a load of 0·75 g fructose per kg body weight, the enhanced postprandial elevation of plasma TAG is mainly explained by a small impact of fructose on insulin compared with glucose, reducing TAG clearance, rather than as a result of new synthesised lipids which appeared to be small. Given the fact that about 50 % of a fructose load is converted into glucose, 25 % into lactate, and approximately 15 % into glycogen, *de novo* lipogenesis is a minor pathway for fructose disposal^(^ ^56^ ^)^. This is in line with the substantial evidence reviewed by Hellerstein *et al.* ^(^ ^130^ ^)^, who summarised the evidence as follows:

(1) After consumption of a normal diet, < 3 % of post-absorptive VLDL was estimated to come from sugar;
(2) In the fed state, < 5–7 % of VLDL post-absorptive comes from sugar;
(3) When given 250 g fructose within 6 h, < 10 % of the fructose load was converted to lipids, equivalent to < 1 g/h in absolute amounts;
(4) Daily overfeeding with 150–200 g fat and 750–1000 g carbohydrates led to a *de novo* synthesis of 5 g fat per d, equivalent to < 3 % of the total fat consumed.

Accordingly, Hellerstein *et al.* ^(^ ^130^ ^)^ concluded that *de novo* net lipogenesis, after fructose or sugar consumption, is in fact very small. The explanation for these observations is that the consumed carbohydrates are primarily cleared from the blood, to be oxidised in energy metabolism and/or stored as glycogen, at the expense of fat oxidation which drops due to lipolysis inhibition by insulin and reduced NEFA availability. Thus, only very small amounts of lipids are synthesised after large fructose, sugar or carbohydrate loads, unless extreme carbohydrate overloading is sustained for several days^(^ ^131^ ^)^.

Very recently, Sun & Empie^(^ ^132^ ^)^ reviewed isotopic tracer studies in human subjects. The authors summarised their findings as follows: ‘Fructose is readily absorbed and its absorption is facilitated by the presence of co-ingested glucose. Sucrose, honey, 50:50 glucose-fructose mixtures and HFCS all appear to be similarly absorbed. Fructose itself is retained by the liver, while glucose is mainly released into the circulation and utilized peripherally. Plasma levels of fructose are an order of magnitude (10–50 folds) lower than circulating glucose, and fructose elicits only a modest insulin response.’ Further, the authors stated that the average oxidation rate of fructose was similar in non-exercising and exercising conditions (45·0 and 48·8 %, respectively). Moreover, they underscored that when fructose is ingested together with glucose, the mean oxidation rate of the mixed sugars increased significantly.

In their review, Sun & Empie^(^ ^132^ ^)^ described the metabolic fate of pure fructose based on several studies. Following 3–6 h after ingestion, on average 41 (sd 10·5) % fructose was converted to glucose. Only a small percentage of ingested fructose ( < 1 %) was directly converted to plasma TAG. Approximately one-quarter of ingested fructose was converted into lactate within a few hours. They discussed further that the observed increases in plasma TAG and *de novo* lipogenesis, as observed in various studies, can arise from both increased lipid synthesis and decreased lipid clearance, and that the relative contributions were not addressed in any detail in the available studies. Furthermore, the fate of fructose ingested together with glucose had received little attention so far. In addition, habitual fructose intake, health status (and more specifically insulin resistance), sex, or ethnic/genetic background were all-important factors that may modulate sugar–lipid relationships but had not yet been adequately investigated.

Accordingly, the influence of fructose consumption on plasma lipids and *de novo* lipogenesis remains controversial and understudied and conclusions that fructose is a liver toxin similar to alcohol are certainly premature.

### Fructose, uric acid and insulin resistance

In 2009, Johnson *et al.* ^(^ ^133^ ^)^ hypothesised that excessive fructose intake (>50 g/d) may be one of the underlying factors in the aetiologies of the metabolic syndrome and type 2 diabetes. The authors suggest that this occurs through mechanisms by which rapidly increased fructose phosphorylation in liver cells results in total adenine nucleotide degradation leading to the liberation of elevated uric acid, leading to higher cardiovascular risk^(^ ^134^ ^)^. In a study of Sánchez-Lozada *et al.* ^(^ ^135^ ^)^, rats were fed either a combination of 30 % fructose and 30 % glucose or 60 % sucrose, while control rats were fed normal rat chow containing 60 % maize starch. Diets containing 30 % of either both free fructose and free glucose, or as the disaccharide sucrose, induced the metabolic syndrome, intra-hepatic accumulation of uric acid and TAG, leading to fatty liver. Relevant for the interpretation of this study is that the level of fructose consumed by the rats was excessive and does not reflect levels consumed by humans.

Another study, by Abdelmalek *et al.* ^(^ ^91^ ^)^, investigated twenty-five diabetic adults receiving an intravenous fructose challenge. Based on their data, the authors concluded that high fructose consumption depletes hepatic ATP and impairs recovery from ATP depletion after an intravenous fructose challenge. This approach, however, relied on the intravenous administration of >25 g of pure fructose (250 mg/kg body weight) within 1 min, resulting in a massive hepatic disposal. Similar ATP depletion has also been observed with large oral fructose load, but led to only small increases in uric acid concentrations in healthy subjects^(^ ^136^ ^)^. However, it has been recently reported that ingestion of even larger amounts of fructose failed to acutely increase uric acid concentration when ingested in split doses throughout several days, suggesting that liver ATP depletion is unlikely to occur with usual patterns of sugar consumption^(^ ^137^ ^)^.

Lin *et al.* ^(^ ^138^ ^)^ also observed that fructose consumption resulted in higher serum uric acid levels in individuals with a BMI of >30 kg/m^2^. Interestingly, their study showed that there was no effect of fructose intake in the subjects with a BMI between 25 and 29 kg/m^2^, although serum uric acid showed a trend to be elevated depending on body-weight status. Moreover, the blood samples were drawn after an overnight fast in the morning, ruling out any postprandial effect of fructose ingestion. Accordingly, the effect on serum uric acid was more likely to be secondary to obesity or the metabolic syndrome than to fructose consumption *per se*. In this study, intake was calculated from food frequency recall, which is known to have a low level of accuracy^(^ ^139^ ^)^. Moreover, food frequency intake data are based on food composition tables that are not controlled for recipe-related changes in food and beverage products on the market. This double chance of error should not be neglected.

Limited data are available on serum uric acid changes after realistic dietary loads of fructose-containing sugars^(^ ^140^ ^)^. For example, Akhavan & Anderson^(^ ^141^ ^)^ tested solutions containing different ratios of glucose and fructose. In their study, overnight-fasted men received a standardised breakfast in the morning. At 4 h later, a 300 kcal (1255 kJ) drink was ingested within 3 min. The solutions were sweetened with either HFCS containing 55 % of fructose, sucrose or the monosaccharide forms of glucose and fructose in specific ratios as follows: 80 % glucose: 20 % fructose (G80:F20), sucrose, G50:F50, G35:F65 and G20:F80. At 75 min, uric acid concentrations were highest after G20:F80. The sucrose and F50:G50 solutions each resulted in significantly lower uric acid concentrations than did the G20:F80 solution, but they did not differ significantly from any other solutions. The uric acid AUC did not differ significantly after the G35:F65, G50:G50 and sucrose solutions. In other research^(^ ^142^ ^)^, only a weak response of serum uric acid to fructose was found.

Very recently, Wang *et al.* ^(^ ^143^ ^)^ conducted a systematic review and meta-analysis of controlled fructose-feeding trials. The authors noted that hyperenergetic supplementation of control diets with excessive fructose (+35 % excess energy, i.e. 213–219 g/d) significantly increased serum uric acid compared with the control diets in non-diabetic participants (mean difference 31·0 (95 % CI 15·4, 46·5) mmol/l). Confounding from excessive energy could not be ruled out in the hyperenergetic trials, because no uric acid-increasing effect of tested fructose, isoenergetically exchanged with other carbohydrates, was noted in either the non-diabetic or diabetic trial.

Zgaga *et al.* ^(^ ^144^ ^)^ recently observed a positive association between plasma uric acid and SSB consumption, but no association with fructose intake, leading the authors to suggest that fructose is not the causal agent underlying the SSB–urate association. In another cross-sectional study^(^ ^145^ ^)^, it was also concluded that higher dietary fructose intake was not associated with a higher hyperuricaemia risk in healthy adults. This is in line with the results of a meta-analysis^(^ ^146^ ^)^ and review^(^ ^147^ ^)^ that refuted the relationship between normal dietary consumption of sugars containing fructose and diabetes.

## Final considerations

As discussed, recent findings suggest that high or excessive fructose intake can induce certain metabolic alterations in both animal and human models. In this respect, thoughts regarding the potential harmfulness of excessive fructose and fructose-containing sugar intakes seem legitimate, especially in view of the high SSB consumption and the burdens of obesity and type 2 diabetes.

Based on the currently available data, however, any statement that ordinary fructose intake is toxic and that consumption of fructose-containing drinks are the leading cause of the global obesity epidemic is not supported by scientific consensus. We wish to highlight the findings of Gibson^(^ ^148^ ^)^, who re-examined the evidence from forty observational and four intervention studies, as well as six reviews. She noted that the totality of the evidence was dominated by American studies and that most studies suggest that the effect of SSB is small except in susceptible individuals, involving genetic predispositions, psychological factors and environmental stimuli^(^ ^149^ ^)^, or at excessive levels of intake (>20 % of total energy). She reported that progress in reaching a definitive conclusion on the role of SSB in obesity is hampered by the paucity of good-quality interventions, which reliably monitor diet and lifestyle and adequately report effect sizes. Of the three long-term (6 months) interventions, one reported a decrease in obesity prevalence but no change in mean BMI and two found a significant impact only among children already overweight at baseline. Of the six reviews, two concluded that the evidence was strong, one that an association was probable, while three described it as inconclusive, equivocal or near zero.

Noteworthy is the study of Pollock *et al.* ^(^ ^150^ ^)^, who observed in adolescents that higher fructose consumption is associated with multiple markers of cardiometabolic risk, but when visceral adipose tissue was included as a covariate, it attenuated these associations and showed that these relationships were mediated by visceral obesity.

Also, Rizkalla^(^ ^27^ ^)^ concluded that ‘no fully relevant data have been presented to account for a direct link between dietary fructose intake and health risk markers’. A re-evaluation of published epidemiological studies concerning the consumption of dietary fructose or mainly HFCS showed that most of these studies have been cross-sectional or based on passive inaccurate surveillance, especially in children and adolescents^(^ ^151^ ^)^, and thus have not established direct causal links. Research evidence of the short- or acute-term satiating power or increasing food intake after fructose consumption as compared with that resulting from normal patterns of sugar consumption, such as sucrose, remains unclear. Further, the negative conclusions regarding fructose have been drawn from studies in rodents or in human subjects attempting to elucidate the mechanisms and biological pathways underlying fructose consumption by using unrealistically high amounts of pure fructose. In this respect, we also want to draw attention to the results of a data analysis by Livesey^(^ ^152^ ^)^ who, based on the data of several large cohorts, concluded as follows:

‘Fructose is proving to have bidirectional effects. At moderate or high doses, an effect on any one marker may be absent or even the opposite of that observed at very high or excessive doses; examples include fasting plasma triglyceride, insulin sensitivity, and the putative marker uric acid. Among markers, changes can be beneficial for some (e.g., glycated hemoglobin at moderate to high fructose intake) but adverse for others (e.g., plasma triglycerides at very high or excessive fructose intake). Evidence on body weight indicates no effect of moderate to high fructose intakes, but information is scarce for high or excessive intakes. The overall balance of such beneficial and adverse effects of fructose is difficult to assess but has important implications for the strength and direction of hypotheses about public health, the relevance of some animal studies, and the interpretation of both interventional and epidemiological studies. By focusing on the adverse effects of very high and excessive doses, we risk not noticing the potential benefits of moderate to higher doses, which might moderate the advent and progress of type-2 diabetes, cardiovascular disease, and might even contribute to longevity.’^(^ ^152^ ^)^

## Conclusion

Through multiple misconceptions about fructose and fructose-containing sugars, a causal role of their intake has been proposed in the aetiology of the global obesity epidemic. However, current evidence on the metabolic effects of fructose, as consumed by the majority of populations, is insufficient to demonstrate such a role in metabolic diseases and the global obesity epidemic.

Given the impact of obesity and related metabolic diseases on health care costs, practical steps to prevent their development are obviously required. Nevertheless, implementing taxes on sugary foods and beverages as suggested is not supported by solid scientific evidence, and can be expected to be largely insufficient to address the whole issue of energy overconsumption^(^ ^153^ ^,^ ^154^ ^)^. In this respect, one may rather aim at reducing the consumption of energy-dense foods, which represent a large panel of sweet and salted foods made largely available in shops, fast foods and restaurants. The food production and service industries would be welcome to play a responsible role by gradually limiting the amount of fat and added sugars in ready-to-eat or to-drink products to reduce energy density. In addition, effective policies that facilitate and promote healthier diets and nutritious food alternatives should be publicly promoted.
